# A transdiagnostic AI-based measure of interpersonal coordination in autism and other conditions

**DOI:** 10.1186/s13229-026-00722-3

**Published:** 2026-05-29

**Authors:** Lisa D. Yankowitz, Casey J. Zampella, Juhi Pandey, G. Keith Bartley, Julia Parish-Morris, Edward S. Brodkin, John D. Herrington, Birkan Tunç, Robert T. Schultz, Evangelos Sariyanidi

**Affiliations:** 1 Center for Autism Research, The Children’s Hospital of Philadelphia, Philadelphia, 19146 USA; 2 Department of Psychiatry, Perelman School of Medicine, University of Pennsylvania, Philadelphia, 19104 USA; 3 Department of Child and Adolescent Psychiatry and Behavioral Sciences, Children’s Hospital of Philadelphia, Philadelphia, PA 19104 USA; 4 Department of Biomedical and Health Informatics, The Children’s Hospital of Philadelphia, Philadelphia, PA 19104 USA; 5 Department of Pediatrics, University of Pennsylvania, Philadelphia, 19104 USA

**Keywords:** Interpersonal coordination, Interpersonal synchrony, Autism, Transdiagnostic

## Abstract

**Background:**

Interpersonal coordination is a fundamental social behavior that has been shown to be reduced in autism, though less is known about other psychiatric conditions. An automated quantitative measure of interpersonal coordination would enhance assessment, diagnosis, and monitoring of treatment-related change in autism and other psychiatric conditions. We introduce and apply a novel AI-based measure (’concurrence’) to quantify and compare nonverbal interpersonal coordination during naturalistic conversation in individuals with and without various psychiatric presentations.

**Methods:**

The primary analysis included 380 12–18-year-olds with neurotypical development (NT), autism (AUT), or other psychiatric conditions (PSY), recorded during videoconference get-to-know-you conversations with a research staff member (‘partner’). Replication analyses included 72 12–18-year-olds with NT or AUT, recorded during face-to-face conversations. A self-supervised AI method (concurrence) was applied to time series data representing facial expressions and head movements of participants and their conversation partners. This yielded interpersonal coordination scores for all participant-partner dyads, which were then compared transdiagnostically. Convergent and discriminant validity were assessed using annotated subsamples from a combined sample of 609 5–52-year-olds. Convergent validity was assessed with measures of social gaze, motor imitation ability, and conversation quality; discriminant validity was assessed with IQ scores.

**Results:**

In the videoconference sample, AUT demonstrated significantly lower interpersonal coordination than PSY (unadjusted Cohen’s *d = 0.46*, *p* < 0.001) and NT (*d =* 1.03, *p* < 0.001), with PSY also lower than NT (*d* = 0.50, *p* < 0.001). The AUT < NT effect was replicated in the face-to-face sample (*d = 0.73*, *p* < 0.05). The group-by-context interaction was nonsignificant (*p* = 0.33), suggesting group differences are robust to recording context. Convergent and discriminant validity was demonstrated through positive associations between interpersonal coordination and mutual social gaze (*r(108)* = 0.46, *p* < 0.0001), gross motor imitation ability (*r(35)* = 0.41, *p* < 0.05), and conversation quality ratings (*r(364)* = 0.34, *p* < 0.0001), but not IQ (*r(367)* = 0.03, *p* = 0.55).

**Limitations:**

Generalizability is limited by sample characteristics including cognitive and verbal ability, age, and sex.

**Conclusions:**

The study demonstrates reduced interpersonal coordination in adolescents with autism and other psychiatric conditions (AUT<PSY<NT) using a novel, transdiagnostic computational measure. This finding replicated across different conversational contexts and the concurrence measure demonstrated convergent and discriminant validity, highlighting its potential as an automated and scalable individual-level measure of social skills.

**Supplementary Information:**

The online version contains supplementary material available at 10.1186/s13229-026-00722-3.

## Background

Interpersonal coordination is the tendency for social partners to spontaneously coordinate their behavior with one another during interactions. This phenomenon occurs unconsciously across social-motor behaviors such as facial expressions [1, 2], body [3] or limb movements [2, 4], and speech behaviors [5]. This coordination of behaviors is a core aspect of social behavior with evolutionarily adaptive functions [6]. Evidence suggests that interpersonal coordination is bidirectionally related to prosocial behavior [7–11] and to judgments of the interaction and interaction partner [12].

Interpersonal coordination is essential for social development. It is present from birth, with neonates synchronizing limb movements to parent speech [13]. Infants rely on synchronous patterns within their social environments to form early bonds, which provide the foundation for a cascade of positive developmental outcomes including self-regulation, symbolic play, theory of mind, empathy, and language [14]. Interpersonal coordination continues to influence social development in childhood and adolescence [15, 16].

Reductions in interpersonal coordination in autism relative to neurotypical development have been repeatedly demonstrated [17, 18], with meta-analysis indicating a large effect size [19]. Autism is defined by social communication differences, and its core diagnostic criteria (i.e., atypical social reciprocity and nonverbal communication) have clear overlap with the construct of interpersonal coordination [20]. Examples of coordination reduced in autism include contagious yawning [21], synchronized chair rocking [22], synchronized movements when assembling a puzzle with an examiner [23], synchrony in head and hand movements [24], and spontaneous matching of social partners’ facial expressions [25, 26].

Reduced interpersonal coordination has also been observed in other psychiatric conditions including depression [27, 28], social anxiety disorder [29], attention-deficit/hyperactivity disorder [30], and schizophrenia [31], but with less evidence available. Direct comparison between autism and other conditions is lacking, leaving the specificity of interpersonal coordination reductions in autism unclear. The mechanisms proposed to impact interpersonal coordination may be transdiagnostic, and include social attention, motivation, and orienting; multisensory and temporal processing; and motor action prediction and performance [17, 32].

Despite its significance, there is no universal operationalized quantitative measure of interpersonal coordination [33]. This greatly hinders reproducibility and the drawing of generalizable conclusions [34]. Coordination can be operationalized narrowly in terms of spontaneous mimicry – behaviors coordinated in time, shape, and form (e.g., reciprocal smiling). However, discrete mimicry events occur infrequently during natural interactions. A more comprehensive definition of interpersonal coordination – any non-conscious co-adaptation of social partners’ behaviors in response to one another over time [3] – is more relevant for examining coordination in naturalistic social contexts, but has proven difficult to measure.

Operationalizing this broader definition of interpersonal coordination typically requires extracting, from video, multiple behavioral time-series signals per social partner (e.g., face or body movements), yet no universally agreed upon analytic approach exists for analyzing these signals. One widely used method in behavioral research is windowed (linear) cross-correlation (WCC) [26, 35–38], which computes the correlation between signals within sliding windows across multiple time lags. In studies of interpersonal coordination in autism, WCC has been applied to time series of motion energy (which quantifies movement based on the number of pixels changing between frames in a video within a specific region of interest) [23, 38, 39], upper body joint pose estimates [40], and magnitude of smiles [26]. While WCC methods have been useful in identifying some interpersonal coordination differences in autism, a general drawback is that they cannot capture coordinated behaviors that differ in form (e.g., linear change in one signal and quadratic change in the other, perhaps smiling in response to nodding) or speed (e.g., a rapid smile in response to a slow one), and thus do not capture the full possible range of interpersonal coordination. Another common approach is relative phase, which is an angle that represents where one person’s movement is within a rhythmic cycle relative to where another movement is in its cycle (e.g., 0° in-phase, truly synchronized; 180° anti-phase, alternating). In autism research, this approach has been applied to chair-rocking [22], pendulum-swinging [41], and body/object imitation tasks [42]. It shares WCC’s limitations of inability to capture different form or speed of behaviors and is only appropriate for behaviors with an oscillatory structure. Wavelet-based methods [43, 44] can overcome some limitations of linear WCC and phase-based approaches by decomposing behavioral signals into component frequencies and assessing phase alignment at different timescales. However, these approaches generally require a priori assumptions about the form of coordination (e.g., in-phase or anti-phase), and investigating coordination across multiple time-frequency bands or wavelet parameters leads to an excessive number of statistical comparisons. The multiple comparison problem is compounded when behavior is encoded with multi-dimensional time series (e.g., dozens of signals representing components of facial expressions), as the number of necessary statistical comparisons grows exponentially. Lastly, AI-based diagnostic classification methods that implicitly quantify interpersonal coordination [37] provide group-level separation (e.g., AUT vs. NT) rather than a score, and thus cannot be used transdiagnostically, which significantly hinders their utility in mental health conditions that are heterogeneous and highly overlapping. Further, when classification is WCC-based [37], the method inherits WCC’s inability to capture non-linear behavioral dependencies. These methods may also fail to truly capture dyadic-level coordination, as autocorrelations within signals can lead to spurious cross-correlation [45]. A robust method for quantifying interpersonal coordination that overcomes these shortcomings is needed to enhance understanding of its developmental and functional importance across psychiatric presentations and to identify social biobehavioral markers.

We recently introduced a novel AI approach [46] (‘concurrence^1^’) that can detect any form of statistical dependence between (multi-dimensional) behavioral signals and thus can comprehensively capture interpersonal coordination. Concurrence leverages self-supervised deep learning to learn non-linear dependencies among signals that differ in form and speed, without requiring a priori information regarding the nature of the coordination. It is a fully automatic approach not requiring user input (e.g., manual annotations). It produces a single summary score (concurrence-based coordination) per interacting dyad that represents the dependencies among multiple behavioral dimensions. Thus, concurrence leverages the full richness of social behavioral signals without loss of statistical power and produces a dimensional score that can be analyzed with traditional statistics to examine group and individual differences, and to monitor change over time.

In the current study, we apply concurrence to study interpersonal coordination among facial expressions and head movements in individuals with a range of psychiatric presentations as they engage in brief, natural dyadic conversations. We hypothesized that concurrence scores would capture transdiagnostic patterns in interpersonal coordination, following the pattern: autism < other psychiatric conditions < neurotypical individuals. We also hypothesized that interpersonal coordination scores would positively correlate with imitation ability, amount of mutual social gaze, and perceived conversation quality (convergent validity), but not with IQ (discriminant validity).

## Methods

**Participants.** Two samples were selected from participants across multiple studies at the Center for Autism Research. Participants in each sample completed a video-recorded semi-structured conversation (see Experimental Tasks). The primary sample (*n* = 380; 12–18 years old) performed the task through videoconferencing. A second, replication sample performed it in person (*n* = 72; 12–18 years old, `face-to-face adolescent sample`); the two participant samples were entirely non-overlapping. Convergent and discriminant validity analyses were conducted on annotated subsamples from the largest available sample (*N* = 609) of participants who completed the task, including participants outside the 12–18-year-old range.

All participants had a clinical evaluation supervised by a licensed clinical psychologist using standardized assessment tools (see Additional File 1 for details) and were classified using best clinical judgment as: AUT (received a diagnosis of autism), PSY (mixed psychiatric group; received a diagnosis of at least one DSM-5 disorder other than autism, with no autism diagnosis), or NT (neurotypical; no DSM-5 diagnoses). Participant information is provided in Table 1, and additional inclusion and exclusion criteria, details about the evaluation, and clinical characteristics are provided in Additional File 1, Tables S1 and S2. 

**Experimental Task.** All participants completed an adapted version of the Contextual Assessment of Social Skills (CASS) [47], a brief (3–5 min), semi-structured “get-to-know-you” conversation with a member of the research staff (‘partner’) while synchronized videos were recorded of both people. Partners were young adult research assistants or college students from the lab whom the participant had never met; they were assigned based on availability. All partners in the videoconferencing sample and a majority in the face-to-face sample were female. Partners were instructed to appear interested and engaged but not carry the conversation (i.e., speak no more than 50% of the time and wait 5 s to re-initiate the conversation after a lapse).

***Face-to-Face.*** Participants and partners were seated approximately 58 inches apart, and the CASS conversation was recorded by two simultaneously recording HD cameras. A single device housing both cameras was placed directly between them and adjusted to chest-level height. Participants were asked to stay seated in their chair facing forward and to ignore the camera as much as possible. Cameras captured videos at 30 or 60 frames per second.

***Videoconferencing.*** Participants and partners completed the CASS via lagless videoconferencing from separate rooms, which allowed for maskless conversation during the COVID-19 pandemic. Participants and partners were each seated in front of a computer monitor and webcam, which were connected directly via cables to prevent lags that can occur with typical web-based teleconferencing platforms. Each individual saw only the other person’s video (i.e., their own video was not displayed on the screen). Participants were seated approximately three feet from the screen, and the height of their chair was adjusted manually to ensure their face was in the frame. Cameras captured videos at 60 frames per second.

### Video pre-processing.

Video trimming excluded the first 10 s and segments beyond 240 s (videoconferencing) or 165 s (face-to-face) to achieve consistent video length within each recording context. Trimmed videos were processed with 3DI, a state-of-the-art automated face analysis algorithm [48]. Using 3DI, time series (frame-by-frame data) of three head orientation/pose parameters (pitch, yaw, roll) and 79 facial expression parameters were extracted for the participant and partner. The expression parameters describe the degree of deviation from a neutral face [48].

### Quantifying interpersonal coordination.

**Fig. 1:**
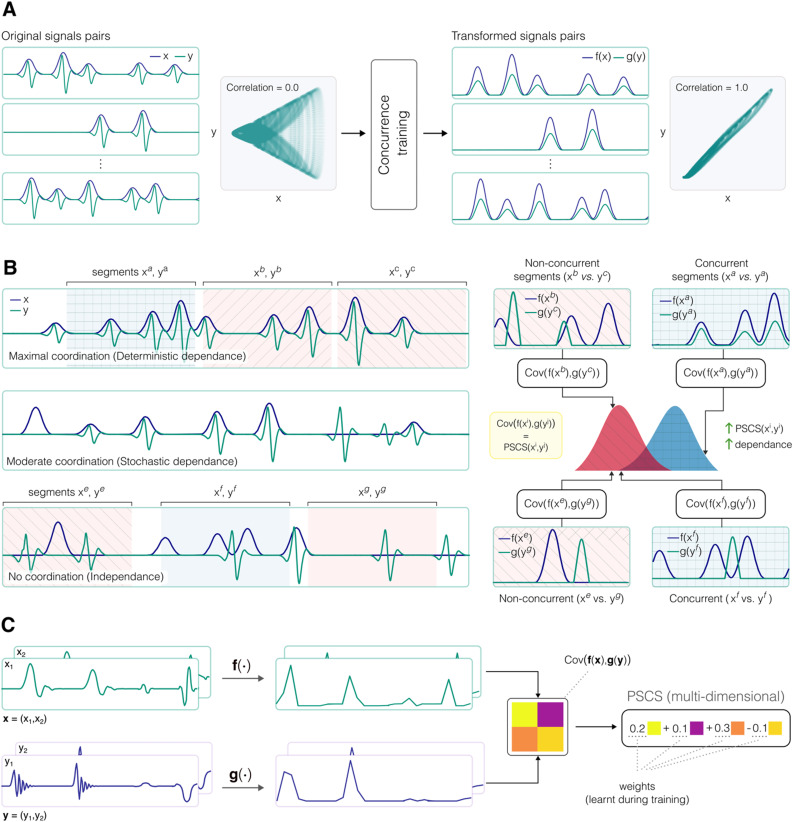
Illustration of the concurrence approach and the computation of the per-segment concurrence score (PSCS). **(A)** Pairs of signals (x, y) that are highly dependent but uncorrelated, as the dependence is non-linear. The dependence can be exposed by finding functions f and g such that the transformed signals f(x) and g(y) are correlated. **(B)** The concurrence approach automatically discovers the functions f and g that expose the (non-linear) dependence by training a deep learning model to classify between concurrent segments (highlighted in blue) and non-concurrent segments (i.e., temporally misaligned; highlighted in red) from the compared signals x and y. This approach is motivated by the fact that the statistical characteristics of concurrent vs. non-concurrent pairs of segments are different only if these segments are extracted from statistically dependent signals. In the illustrated signals with maximal coordination (deterministic dependence), the covariance (i.e., PSCS) computed from the concurrent segments is high, indicating strong dependence, whereas the one computed from non-concurrent signals is low. For independent signals, this covariance is low regardless of whether the segments are concurrent or not. **(C)** The computation of PSCS multiple (i.e., multi-dimensional) signals **x** and **y**. The functions **f** and **g** have a multi-dimensional output in this case, and the covariance between the transformed signals **f(x)** and **g(y)** becomes a matrix. The PSCS is computed as the weighted average of the entries in this covariance matrix, with the weights learned by the deep learning algorithm

A nonverbal interpersonal coordination score was generated for each participant using the ‘concurrence’ method [46], as illustrated in Fig. 1. Conceptually, concurrence operationalizes interpersonal coordination as any statistical dependence between two individual’s behavior. To do this, concurrence compares observed interaction data to an interaction-specific null model constructed from the same dyad. The null comparison is created directly from the interaction itself by pairing short segments from the two individuals that occurred at different moments in time, effectively generating shuffled “pseudo-interactions.” These preserve each person’s individual behavior (e.g., expressiveness, variability) while destroying their moment-to-moment coupling. When the real, temporally-aligned segment pairs have stronger between-person relationships than the pseudo-pairs, it indicates that the partners are coordinating their behaviors.

This framework readily captures well-known forms of interpersonal coordination that manifest as linear relationships. For example, in the simple case of smile mimicry, one person’s smiling is associated with increased smiling in their partner, producing a positive correlation between corresponding facial expression signals in temporally aligned segments. In contrast, facial expression signals in the misaligned segments of the null model are expected to be uncorrelated because temporal alignment is disrupted.

Critically, concurrence captures temporal relationships between partners’ behaviors even when those behaviors do not produce simple linear correlations. For example, in a conversation, one person may nod their head, and the other may respond by changing their facial expression or making a different head movement. Although these behaviors are not the same, and their time series are not correlated, they may show a reliable temporal relationship if they are repeated over the course of the conversation. That is, their relationship may only become statistically apparent after appropriate mathematical transformations (e.g., squaring a time series or applying Fourier transform).

Formally, concurrence addresses this problem by leveraging the theorem that if two time series x[t] and y[t] (e.g., time series of head movements from person one and two) are highly dependent but uncorrelated, then the dependence can be detected by finding transformations (i.e., functions) f and g such that the transformed time series f(x) and g(y) are linearly correlated. Operationalizing this theorem has previously been difficult, as the transformations that expose non-linear dependence are typically unknown and cannot be determined manually. The concurrence method addresses this problem by learning the functions f and g directly from the data using a deep learning network (Fig. 1A) [49]. The strength of the concurrence approach is that it generalizes from simple linear relationships (e.g., smile mimicry) to non-linear relationships (e.g., nodding-expression coupling) by automatically learning transformations of input signals that reveal the non-linear dependencies, effectively converting them into correlated representations (Fig. 1A-B).

Specifically, the model is trained to distinguish between concurrent (temporally aligned) and non-concurrent (misaligned) segments pairs sampled from the same dyad. This self-supervised objective leverages the fact that differences between these two types of segment pairs arise only when the underlying signals are dependent [46]. The covariance between (transformed) segments from dependent and only dependent signals will be high if the segments are concurrent, and low if they are not (Fig. 1B). Since the extraction of concurrent or non-concurrent segments from pairs of signals can be done in a fully automated manner (i.e., self-supervision), the concurrence approach exposes possible non-linear behavioral dependencies without requiring any user input, including diagnostic information, resulting in a naturally transdiagnostic measure.

After transformation, the degree of statistical dependence between segment pairs (participant and partner) is quantified using the per-segment concurrence score (PSCS, Fig. 1B). PSCS is obtained by first applying the learned mathematical transformations to the input segments, then computing the covariance matrix between the transformed segments, and finally calculating a weighted average of the entries of the covariance matrix, with weights learned by the deep-learning algorithm (Fig. 1C). Higher PSCS values indicate stronger dependence. The mean of all PSCSs across the conversation is interpreted as the participant’s interpersonal coordination score.

In this study, the input for concurrence was the time series of the three head pose and 79 facial expression parameters described above, capturing the way a participant’s and partner’s head movements and facial expressions coordinate with one another. Since deep learning can be sensitive to parameter initialization, we trained ten different models with different (random) initializations and averaged over the interpersonal coordination scores provided by the ten networks. To make comparisons across samples commensurate, the training of the machine learning model needed for the coordination scores was performed on the combined sample (i.e., videoconferencing and face-to-face). This is permissible because concurrence is diagnosis-agnostic (i.e., it learns temporal concurrence, not diagnostic labels). Concurrence requires the segment size (i.e., time window) $\:w$ to be specified a priori. Our main analyses are conducted with $\:w=4$ seconds [50], with results for additional time windows (2–32 s) provided in Additional File 1.

To assess modality-specific contributions to overall coordination, we recomputed the concurrence-based coordination metric under two additional conditions: once using only the 3 head-pose parameters and once using only the 79 expression parameters. As with the full metric, PSCS values were averaged to obtain a ‘head-pose coordination’ and ‘expression coordination’ score per participant.

To assess test-retest reliability and partner effects, the concurrence method was calculated on a set of 267 participants who were part of the main samples and also had an available video of a second CASS with a different partner. Concurrence was trained on the full set of both administrations, and a coordination score was calculated for each dyad at each administration.

As an initial exploration of the effect of time on coordination over the course of the conversation, two coordination scores were calculated for the videoconferencing sample by averaging the first half of the PSCS values (first half coordination, approximately seconds 10–125 of the CASS) and the second half of the PSCS values (second-half coordination, approximately seconds 125–240).

### Assessment of skills associated with interpersonal coordination.

Convergent and discriminant validity of the coordination score was assessed through correlations with putatively related (conversation quality, social gaze, and gross motor imitation skills) and unrelated (IQ) scores, which were available in partially overlapping subsets of the samples.

The Conversation Rating Scale (CRS) [47] was originally developed for use with the CASS based on well-validated interpersonal communication scales. The original scale included five items rating the other person’s interest, boredom, and friendliness, and the sense of conversational flow and distance on a 7-point Likert scale. A 6-item adapted version was used with the face-to-face sample (*n* = 142), with an additional question about eye contact. A 15-item adapted version was used in the videoconferencing sample (*n* = 366), with additional questions about comfort, awkwardness, facial expressions, and synchrony, among others. See Additional File 1 for the full set of items in each version. In both samples, the conversational partner completed the CRS immediately following the interaction. Both versions showed good internal reliability in these samples (15-item version Cronbach’s α = 0.96, 6-item version α = 0.91).

A subset (*n* = 37; ages 9–17 years) of participants from the face-to-face sample also completed a gross motor imitation task, where they imitated a sequence of body movements demonstrated on a screen by a male actor [51]. Imitation scores were generated for each participant by human coding (see Additional File 1 for details).

An AI-derived mutual social gaze score [52] was also calculated for a subset (*n* = 110; ages 7–49 years) of the face-to-face sample. This score was calculated as the proportion of frames in which both the participant and partner were labeled by the AI algorithm as looking at each other.

Cognitive ability was assessed for each participant with a standardized measure [53–56] (see Additional File 1); Standard Scores are collectively referred to as ‘IQ,’ and were available for *n* = 369 videoconference participants, and *n* = 214 face-to-face participants.

**Statistical analysis.** The computed concurrence (interpersonal coordination) score was used as the dependent variable in linear models. Two identical models were evaluated separately for the videoconferencing and face-to-face adolescent samples, each of the form coordination ~ group + sex + age. Effect size is reported as unstandardized regression coefficients (*B*). Cohen’s *d* from an equivalent t-test is provided as a standardized effect size, which is not adjusted for covariates. Pearson’s correlation was used to assess convergent and discriminant validity. Because *n* = 37 face-to-face participants had a first-degree relative within the sample, a sensitivity analysis was conducted excluding first-degree relatives, with minimal change in effect sizes (see Additional File 1).

## Results

### Autistic adolescents demonstrate lower coordination with a conversational partner than those with mixed psychiatric conditions or typical development

**Fig. 2:**
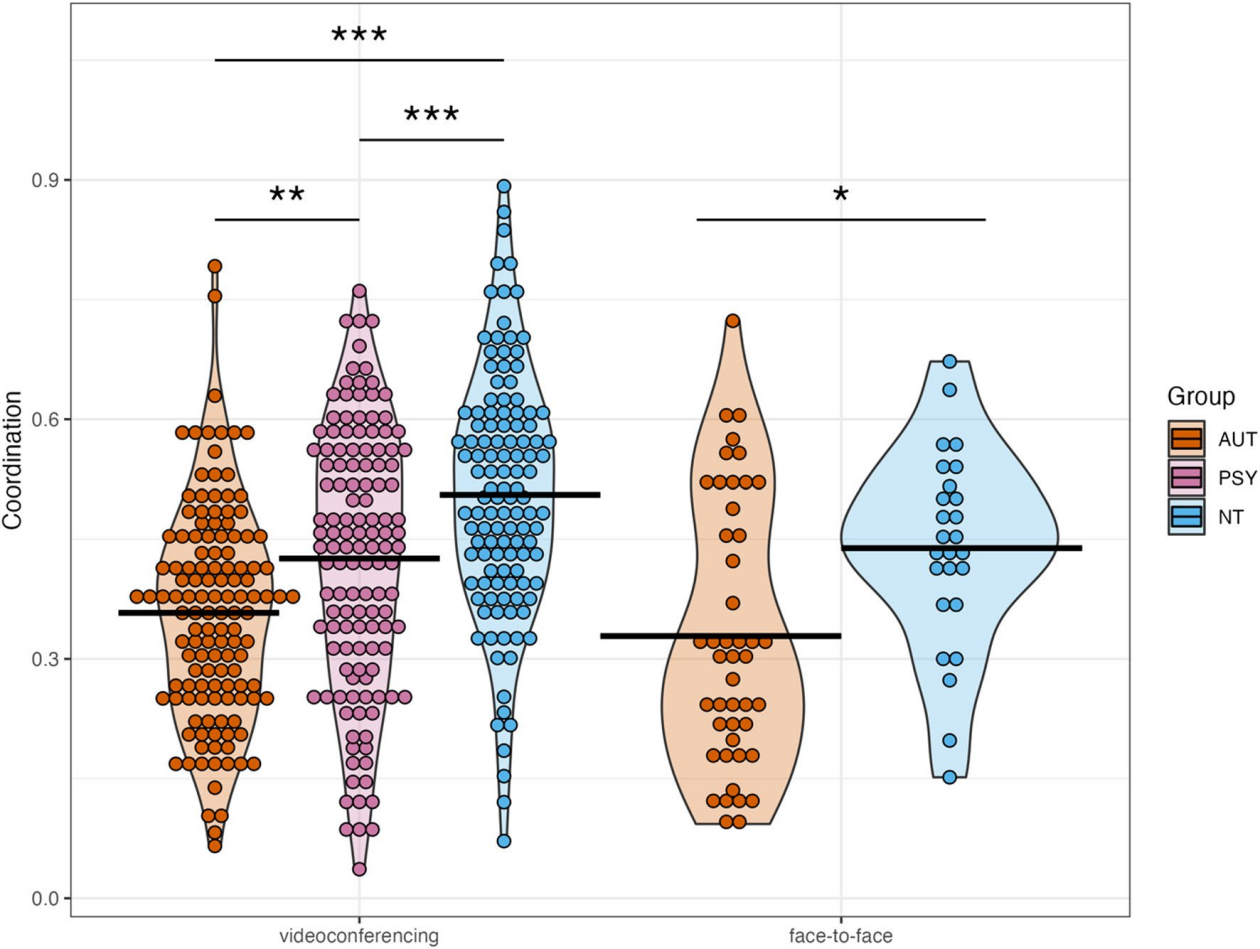
Violin plots for adolescents in the videoconferencing context (left) and the face-to-face context (right). Solid lines indicate the group median. *p < 0.05, **p < 0.01, ***p < 0.001, from linear models controlling for age, sex, and IQ

In a linear model controlling for age, sex, and cognitive ability, the AUT group demonstrated significantly lower interpersonal coordination compared to the NT (unstandardized beta *B* = 0.14, *p* < 0.0001) and PSY (*B* = 0.071, *p* < 0.001) groups in the videoconferencing conversation (Fig. 2; Table 2). Unadjusted for covariates, the effect size of the reduced coordination in AUT was large compared to NT (Cohen’s *d* = 1.03) and small-to-medium compared to PSY (*d* = 0.46). When the reference group was rotated, the PSY group demonstrated significantly lower coordination than the NT group (*B* = 0.073, *p* < 0.001), with medium effect size (*d* = 0.5). Thus, ranking of the degree of coordination was AUT < PSY< NT. This pattern was robust to the choice of time window used to calculate concurrence from 4 to 32 s (see Additional File 1, Figure S1).

### Reduced coordination in autism replicates and is robust to recording context

The effect of reduced interpersonal coordination in AUT compared to NT was replicated in the face-to-face sample of adolescents (*B* = 0.12, *p* < 0.05), with a medium-to-large effect size (*d* = 0.73, Fig. 2). Analysis of the full age range in the face-to-face sample (5–52 years) and sensitivity analyses removing first-degree relatives from the adolescent sample are presented in Figures S3-4 and Tables S3-4. Although qualitatively greater coordination is observed in NT compared to AUT in the full age range, the difference was not significant (*B* = 0.031, *p* = 0.17), and there was a significant effect of age (*B* = 0.0048, *p* < 0.001), suggesting developmental changes in coordination over this wide age range.

To evaluate the impact of recording context, the videoconferencing and face-to-face samples were matched on group proportion (AUT/NT), age, sex, and cognitive ability; a linear model assessed each of those terms, context, and the group-by-context interaction (Figure S2, Additional File 1). The group-by-context interaction was not significant (*B* = 0.054, *p* = 0.33), indicating that the magnitude of the AUT versus NT effect did not differ significantly based on set up. The interaction term was removed from the model for interpretability of main effects. The effect of group was significant (*B* = 0.12, *p* < 0.0001) indicating that NT demonstrated higher coordination than AUT across both contexts. The effect of context was not significant (*B* = 0.033, *p* = 0.20), indicating that interpersonal coordination was similar across contexts.

### Sex does not moderate differences in coordination

When a group-by-sex interaction term was added to the linear model predicting interpersonal coordination, it was not significant in either the videoconferencing (Sex*NT *B* = -0.038, *p* = 0.31, Sex*PSY *B* = -0.030, *p* = 0.43), or the face-to-face adolescent sample (*B* = -0.10, *p* = 0.21). Across diagnostic groups, there was a significant main effect of sex in the videoconferencing sample, with females showing higher coordination than males (*B* = 0.08, *p* < 0.0001), but this effect was not significant in the face-to-face adolescent sample (*B* = 0.007, *p* = 0.87).

### Convergent and discriminant validity

**Fig. 3:**
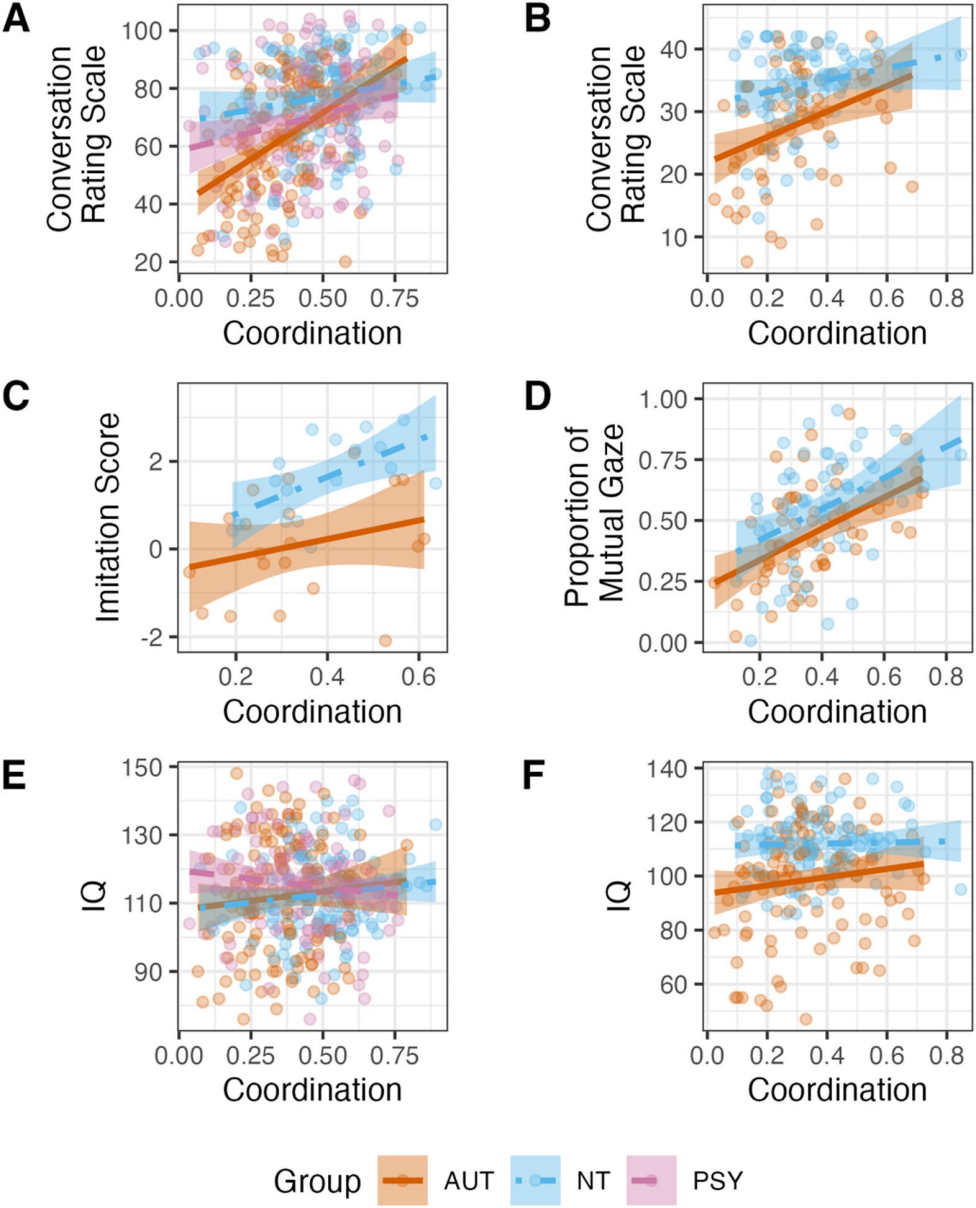
Scatterplots demonstrating convergent validity between coordination and: the conversation rating scale in the videoconferencing (**A**) and face-to-face (**B**) samples; gross motor imitation in a subset of the face-to-face sample (**C**), and proportion of mutual gaze between the Participant and Partner during the interaction in a subset of the face-to-face sample (**D**); as well as discriminant validity between coordination and IQ in the videoconferencing (**E**) and face-to-face (**F**) samples

In the videoconferencing sample, interpersonal coordination was significantly associated with the partners’ ratings of conversation quality (CRS scores, 15-item version) across all groups (*r(364)* = 0.34, *p* < 0.001) and within AUT (*r(122)* = 0.43, *p* < 0.001, Fig. 3A). This was replicated in the face-to-face sample using the 6-item version of the CRS (all groups *r(140)* = 0.30, *p* < 0.001, AUT only *r(67)* = 0.36, *p* < 0.01, Fig. 3B).

In the subset of the face-to-face sample with mutual social gaze scores, interpersonal coordination and mutual social gaze were correlated at *r(108)* = 0.46 (*p* < 0.0001, Fig. 3D).

In the subset of the face-to-face sample with gross motor imitation scores, interpersonal coordination and gross motor imitation were correlated at *r(35)* = 0.41, *p* < 0.05 (Fig. 3C).

Interpersonal coordination scores were not significantly correlated with IQ estimates in either the videoconferencing (*r(367)* = 0.031, *p* = 0.55, Fig. 3E) or face-to-face samples (*r(212)* = 0.13, *p* = 0.067, Fig 3F).

### Within-conversation stability of interpersonal coordination

Across all groups, the correlation between the coordination scores from the first and second halves of the videoconference conversations was *r* = 0.74 (*p* < 0.0001, 95% CI 0.69–0.78). There was a small but significant difference in mean coordination scores between the first and second half (first half *M* = 0.44, *SD* = 0.18, second half *M* = 0.42, *SD* = 0.17, *t*(379) = 3.7, *p* < 0.001, Cohen’s *d* = 0.14). Group (AUT, PSY, NT) did not significantly predict the difference between first- and second-half coordination using ANOVA (*p* = 0.29), indicating that while coordination scores overall tended to decrease between the first and second half of the conversation, this change was not related to diagnostic group membership.

### Contributions of expression and head-pose to coordination

**Fig. 4:**
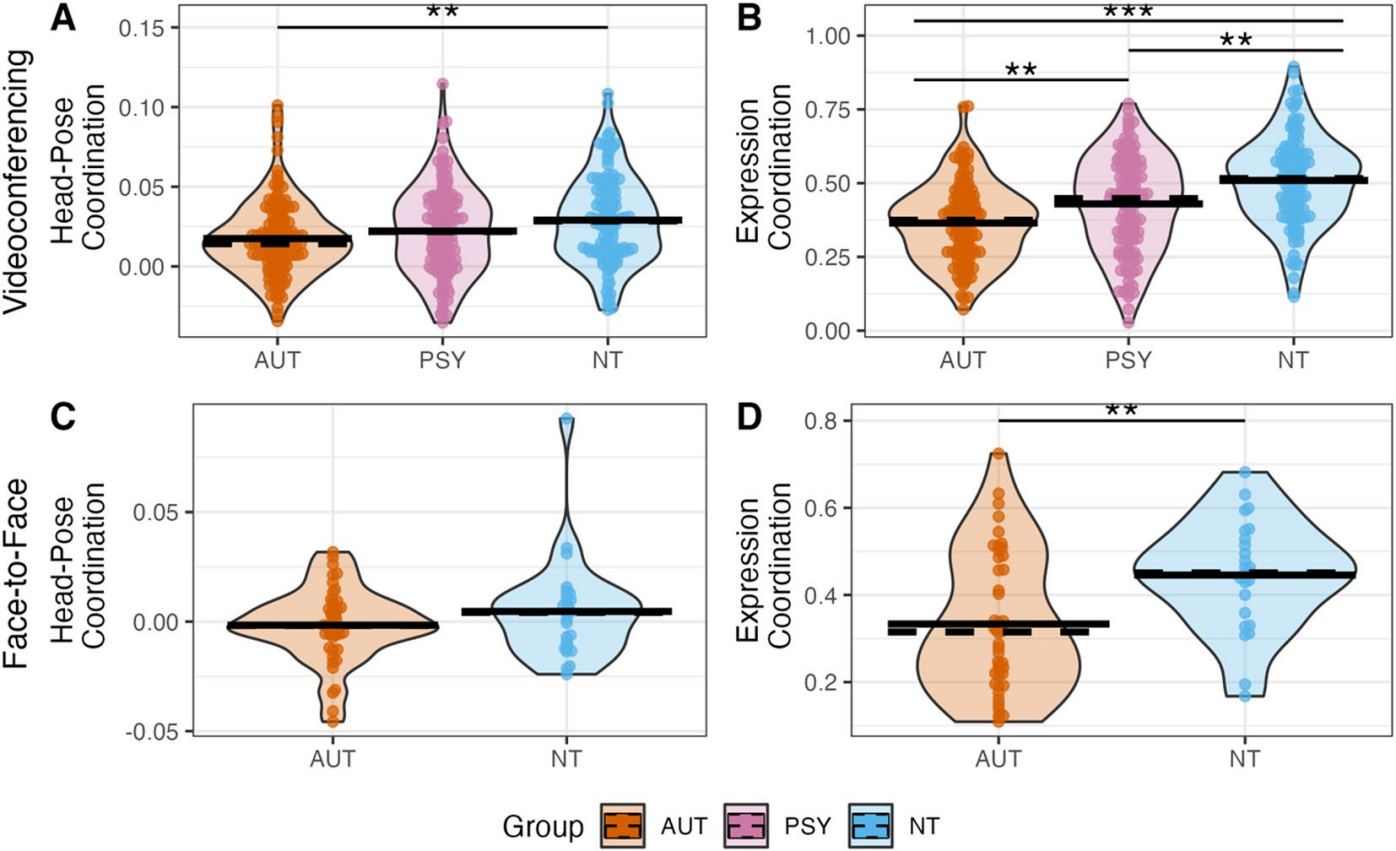
Violin plots showing head-pose coordination (**A**,** C**) and expression coordination (**B**,** D**) in the videoconferencing (**A**,** B**), and face-to-face adolescent (**C**,** D**) samples

Separate head-pose coordination and expression coordination scores qualitatively followed the same group difference pattern as the overall coordination score (AUT < PSY < NT) across the videoconferencing and face-to-face adolescent samples, as shown in Fig. 4. Using linear models controlling for age, sex, and IQ (Tables S5-S6), expression coordination closely matched the magnitude of group differences observed in overall coordination (videoconferencing AUT < NT *B* = 0.14, *p* < 0.001; AUT < PSY *B* = 0.07, *p* < 0.001; PSY < NT *B* = 0.07, *p* < 0.001; face-to-face adolescent AUT < NT *B* = 0.12, *p* < 0.01). In contrast, head-pose coordination differences were smaller and did not all reach significance (videoconferencing AUT < NT *B* = 0.01, *p* < 0.001; AUT < PSY *B =* 0.01, *p* = 0.053; PSY < NT *B* = 0.006, *p* = 0.09; face-to-face adolescent AUT < NT *B* = 0.01, *p* < 0.066). A Shapley regression was used to estimate the relative importance of expression coordination and head-pose coordination in predicting overall coordination. The model explained 99.07% of variance, with expression coordination contributing 0.878 of total variance (88.6% of explained variance) and head-pose coordination contributing 0.113 (11.4% of explained variance).

### Partner effects and reliability of coordination across conversations

A cross-classified linear mixed effects model was fit for the set of concurrence scores generated from the subset of participants who had a second available video of the CASS (*n* = 267), with a fixed effect for administration (first or second), and random effects for participant ID and partner ID. Variance decomposition indicated that 54.5% of the residual variance in the coordination score was attributable to stable between-participant differences, whereas between partner effects accounted for only 6.7%, with the remaining 38.7% within-pair residual variation. Based on these components, the model-based reliability of a single conversation was 0.55. The reliability of participant scores averaged across two conversations was 0.71, estimated as: between-participant variance ÷ [between-participant variance + (between-partner variance + residual variance/2)].

## Discussion

We demonstrate reduced nonverbal interpersonal coordination in autism and other psychiatric conditions using a novel, fully-automated approach for quantifying interpersonal coordination in a naturalistic, generalizable social task. As hypothesized, and consistent with prior evidence [17, 19], autistic adolescents demonstrated significantly reduced interpersonal coordination with conversation partners relative to neurotypical peers, replicated in two separate samples within different contexts (face-to-face and videoconferencing). Autism is a social condition – its essence is not in isolated individual behavior, but in interactions with others [57]. We demonstrate that a principled AI approach can capture dyadic behavioral measurements and create a quantitative score for each participant. Interpersonal coordination is widely recognized as a foundational social construct, with direct relevance to autism as both an outcome and a potential mechanism. Disruptions in other underlying domains (e.g., social attention, motor skills, perceptual processing) may result in reduced coordination [17, 32], and reduced coordination may in turn result in lost opportunities for establishing social-emotional reciprocity, rapport, and relationships [58]. The ability to measure interpersonal coordination automatically and precisely across contexts unlocks its potential as both an intervention target and a bio-behavioral marker for detection and measurement of intervention change, among other uses.

Adolescents with other psychiatric conditions demonstrated a level of interpersonal coordination falling between autistic and neurotypical peers. This lends support for interpersonal coordination as a dimensional construct with relevance across mental health presentations, particularly but not uniquely affected in autism. Most research on interpersonal coordination has been narrowly focused within and between diagnostic groups; this work addresses that gap by providing a direct comparison between autism and other psychiatric conditions. Our findings show that interpersonal coordination is lower in autism than in other psychiatric conditions, but also emphasize the need to examine interpersonal coordination transdiagnostically, e.g., as a subdomain within the social processes domain of the Research Domain Criteria framework [59]. Since concurrence is natively dimensional, it can be used in future behavioral studies to more precisely characterize dyadic social behaviors across conditions, assess treatment-related change, and identify subtypes within autism, and can be used in future genetic studies to identify underlying mechanisms of coordination.

Examination of separate head-pose coordination and expression coordination scores indicated that expression coordination closely mirrored group differences observed in the overall (combined orientation and expression) coordination score. Shapley relative importance analysis attributed 89% of explained variance in overall coordination to expression coordination, and 11% to head-pose coordination. These convergent findings suggest that, while there was evidence for head-pose as a rich communication cue that contributes to coordination and demonstrates group differences, coordination of facial expressions was the dominant driver of observed coordination differences. The concurrence method can be applied in future studies to quantify coordination within and across other behavior domains beyond expression and head-pose, such as gesture, eye gaze, and speech, as it does not require any a priori assumptions about the nature of potential statistical relationships, and therefore can be applied to detect coordination across any quantifiable behaviors. Indeed, our group has demonstrated the validity of the concurrence method for diverse bio-behavioral applications such as detecting relationships between respiration and electrocardiogram signals, and calculating functional brain connectivity from fMRI signals [46].

Group differences in interpersonal coordination were robust to conversational context (face-to-face versus videoconferencing), suggesting that our concurrence method is flexible and generalizable, and that it could be applied remotely via telehealth to assess a fundamental, transdiagnostic social skill. Likewise, while there were small differences in coordination between the first and second half of the conversation, group differences were equivalent in both halves, suggesting that shorter conversations might be sufficient to detect group differences. Examination of pairs of conversations with different partners indicated that stable participant effects account for much more of the variance (55%) in coordination scores than partner effects (7%), and that averaging across two conversations gives a strong boost to reliability (from 0.55 to 0.71). Together, these results imply that if the goal is to measure stable participant traits contributing to coordination, measuring shorter conversations with more partners would yield more reliable results than longer conversations with a single partner. Future work should directly test this, as well as assess reliability in the setting of remote (i.e., in-home) videoconferencing to determine if factors unique to that setting (e.g., home context, noise in video data [e.g., connectivity issues]) impact reliability of coordination measurement.

Four results support the interpersonal coordination score’s convergent and discriminant validity, key components of construct validity. First, consistent with established relationships between interpersonal coordination and judgments of interaction quality [12], higher coordination was associated positively with post-conversation ratings of the interaction quality from the conversational partner (CRS score). This finding suggests that, within the range of naturally occurring behavior expressed during these conversations, higher levels of interpersonal coordination are associated with more positively perceived interactions. Second, social attention is a necessary substrate for interpersonal coordination [32], and we found that higher coordination was significantly associated with the dyad’s mutual social gaze. Third, nonverbal interpersonal coordination likely rests upon core motor imitation skills, including perceiving, planning, and executing motor actions [32]; in our sample, higher coordination was indeed associated with better gross motor imitation ability. On the other hand, interpersonal coordination has been shown to not correlate with IQ [41, 60], and coordination was not associated with IQ in either of our samples, which had predominantly average-to-above average IQ. Further psychometric exploration should focus on generalizability and test-retest reliability across cognitive and language levels, as well as ages.

Future research can extend the present work by applying the concurrence method to examine factors that impact interpersonal coordination in autism, including interaction context and partner characteristics. For example, the double empathy problem suggests that social difficulties in autistic/non-autistic interactions people arise from a bidirectional mismatch in communication styles [61, 62]. Because all partners in the current study were non-autistic, a future comparison of coordination across autistic/autistic, autistic/non-autistic, and non-autistic/non-autistic dyads would help clarify how partners’ neurotypes influence person’s coordination. Additional partner characteristics (e.g., age, race/ethnicity, familiarity, and sex/gender [discussed below]) and variation in conversational (e.g., get-to-know-you, goal-oriented, emotionally valanced) and environmental contexts (e.g., lab- or home-based, quiet or noisy) would further elucidate how coordination unfolds in everyday interactions.

## Limitations

This study has several limitations which should be addressed through future research. The present sample included only individuals with sufficient spontaneous speech to complete a 3–5-minute conversation, which does not represent the full autism spectrum. Future studies can apply the concurrence method to head-pose and expression features extracted from videos of people engaged in interactions without spoken conversation. This study was primarily focused on adolescents, and analysis of the full age range of the face-to-face sample (5–52 years) suggested developmental effects across this wide age range; future work should examine other developmental ranges, including very young children and adults. Our findings were inconsistent with respect to the effect of sex on interpersonal coordination, but interpretations regarding sex differences are limited by small samples. Given that social presentations vary by sex/gender in neurotypical development and in autism, it will be important to further investigate the influence of sex/gender in both participants and conversational partners, as well as other characteristics of the conversational partner. The comparison of contexts (videoconferencing and face-to-face) was a between-subjects design and provided evidence that group effects can be detected with similar magnitude across contexts; a within-subjects design will be needed to directly compare coordination across these contexts. Analysis of temporal dynamics of coordination in the present study was coarse-grained and basic, and future work will apply more sophisticated methods to understand how nonverbal coordination unfolds over a conversation and in relation to speech behavior. Future work will apply the concurrence method within and between behavioral modalities to further elucidate mechanisms of interpersonal coordination.

## Conclusions

For the first time, we provide a dimensional, automated measure of nonverbal interpersonal coordination, derived from naturalistic social interactions, and examine it transdiagnostically and in relation to other core social constructs. Findings across two conversational contexts augment existing evidence of reduced interpersonal coordination in autism and demonstrate significant but less pronounced reductions in other psychiatric conditions. This measure has good psychometric properties, including convergent and discriminant validity and test-retest reliability when averaged over two conversations. Our analyses demonstrate the flexibility of the concurrence method for quantifying different behavioral components of coordination (e.g., expression and pose). These results suggest that automatically-measured interpersonal coordination can serve as a reproducible behavioral marker of social communication differences in autism, and establish a methodological foundation for evaluating coordination in future longitudinal, mechanistic, and intervention-focused research.

**Table 1:** Participant characteristics from the primary and replication samples. characteristics of additional samples are provided in Tables S1 and S2, Additional File 1.

Primary sample: videoconferencing				
	AUT (*N* = 130)	NT (*N* = 120)	PSY (*N* = 130)	*P*-value
**Age**				
Mean (SD)	15.2 (1.67)	14.6 (1.70)	15.1 (1.82)	0.0217
Median [Min, Max]	15.3 [12.3, 18.3]	14.5 [12.0, 18.0]	14.6 [12.4, 18.3]	
**Sex**				
Female	52 (40.0%)	63 (52.5%)	55 (42.3%)	0.11
Male	78 (60.0%)	57 (47.5%)	75 (57.7%)	
**IQ**				
Mean (SD)	112 (16.9)	113 (12.1)	115 (13.8)	0.123
Median [Min, Max]	112 [76.0, 148]	113 [82.0, 140]	117 [76.0, 146]	
Missing	4 (3.1%)	3 (2.5%)	4 (3.1%)	
**SRS-2**				
Mean (SD)	70.3 (11.7)	44.0 (5.71)	55.1 (12.0)	< 0.001
Median [Min, Max]	69.5 [45.0, 101]	43.0 [37.0, 77.0]	52.0 [38.0, 97.0]	
Missing	2 (1.5%)	1 (0.8%)	0 (0%)	
**Race**				
American Indian or Alaska Native	1 (0.8%)	0 (0%)	0 (0%)	0.252
Asian	5 (3.8%)	4 (3.3%)	3 (2.3%)	
Black or African American	19 (14.6%)	21 (17.5%)	8 (6.2%)	
More than one race	4 (3.1%)	8 (6.7%)	9 (6.9%)	
Other	5 (3.8%)	4 (3.3%)	3 (2.3%)	
Unknown/Not Reported	1 (0.8%)	1 (0.8%)	0 (0%)	
White	95 (73.1%)	82 (68.3%)	107 (82.3%)	
**Ethnicity**				
Hispanic or Latino	7 (5.4%)	10 (8.3%)	11 (8.5%)	0.503
Not Hispanic or Latino	123 (94.6%)	109 (90.8%)	119 (91.5%)	
Unknown/Not Reported	0 (0%)	1 (0.8%)	0 (0%)	

Replication sample: face-to-face adolescent			
	AUT (*N* = 46)	NT (*N* = 26)	*P*-value
**Age**			
Mean (SD)	15.4 (2.08)	14.1 (1.50)	0.00412
Median [Min, Max]	15.7 [12.0, 18.7]	13.7 [12.3, 17.9]	
**Sex**			
Female	11 (23.9%)	13 (50.0%)	0.046
Male	35 (76.1%)	13 (50.0%)	
**IQ**			
Mean (SD)	94.0 (22.5)	110 (10.5)	< 0.001
Median [Min, Max]	100 [47.0, 136]	108 [94.0, 136]	
Missing	0 (0%)	3 (11.5%)	
**SRS-2**			
Mean (SD)	65.5 (9.16)	44.5 (4.39)	< 0.001
Median [Min, Max]	66.0 [48.0, 81.0]	44.0 [39.0, 53.0]	
Missing	18 (39.1%)	15 (57.7%)	
**Race**			
Black or African American	5 (10.9%)	2 (7.7%)	0.131
More than one race	4 (8.7%)	4 (15.4%)	
Other	1 (2.2%)	0 (0%)	
White	36 (78.3%)	17 (65.4%)	
Unknown/Not Reported	0 (0%)	3 (11.5%)	
**Ethnicity**			
Hispanic or Latino	3 (6.5%)	0 (0%)	0.125
Not Hispanic or Latino	41 (89.1%)	22 (84.6%)	
Unknown/Not Reported	2 (4.3%)	4 (15.4%)	
	**AUT** **(** ***N*** **= 46)**	**NT** **(** ***N*** **= 26)**	**P-value**
**Age**			
Mean (SD)	15.4 (2.08)	14.1 (1.50)	0.00412
Median [Min, Max]	15.7 [12.0, 18.7]	13.7 [12.3, 17.9]	
**Sex**			
Female	11 (23.9%)	13 (50.0%)	0.046
Male	35 (76.1%)	13 (50.0%)	
**IQ**			
Mean (SD)	94.0 (22.5)	110 (10.5)	< 0.001
Median [Min, Max]	100 [47.0, 136]	108 [94.0, 136]	
Missing	0 (0%)	3 (11.5%)	
**SRS-2**			
Mean (SD)	65.5 (9.16)	44.5 (4.39)	< 0.001
Median [Min, Max]	66.0 [48.0, 81.0]	44.0 [39.0, 53.0]	
Missing	18 (39.1%)	15 (57.7%)	
**Race**			
Black or African American	5 (10.9%)	2 (7.7%)	0.131
More than one race	4 (8.7%)	4 (15.4%)	
Other	1 (2.2%)	0 (0%)	
White	36 (78.3%)	17 (65.4%)	
Unknown/Not Reported	0 (0%)	3 (11.5%)	
**Ethnicity**			
Hispanic or Latino	3 (6.5%)	0 (0%)	0.125
Not Hispanic or Latino	41 (89.1%)	22 (84.6%)	
Unknown/Not Reported	2 (4.3%)	4 (15.4%)	

**Table 2:** Linear models predicting coordination in the primary sample (videoconferencing) and the replication sample (face-to-face adolescents)

Term	Primary: Videoconferencing			Replication: Face-to-Face Adolescent		
	*B*	95% CI	*P* value	*B*	95% CI	*P* value
**Group**						
AUT	—	—		—	—	
NT	0.14	0.11, 0.18	< 0.001	0.12	0.03, 0.20	0.012
PSY	0.07	0.04, 0.11	< 0.001	—	—	
**Sex**						
Female	—	—		—	—	
Male	-0.08	-0.11, -0.05	< 0.001	0.01	-0.07, 0.09	0.872
**Age**	0.01	0.00, 0.02	0.04	0.02	0.00, 0.04	0.048
**IQ**	0	0.00, 0.00	0.542	0	0.00, 0.00	0.204

Abbreviation: *B* = unstandardized coefficient. CI = Confidence Interval

## Supplementary Information

Below is the link to the electronic supplementary material.

Supplementary Material 1

## Data Availability

De-identified data available upon reasonable request to the corresponding author.

## Footnotes

1 Open-source code for the concurrence method is available at https://github.com/sariyanidi/Concurrence.

## Abbreviations

AUT: Autism
CRS: Conversation Rating Scale
NT: Neurotypical
PSY: Mixed Psychiatric
WCC: Windowed Cross-Correlation
