# Supplementary material for: A transdiagnostic AI-based measure of interpersonal coordination in autism and other conditions
Source: Mol Autism. 2026 May 29;17:27. doi: 10.1186/s13229-026-00722-3 (PMC13277197; doi:10.1186/s13229-026-00722-3)
Supplement: Supplementary file 1 — Supplementary Material 1 [file 13229_2026_722_MOESM1_ESM.docx]

A Transdiagnostic AI-Based Measure of Interpersonal Coordination in Autism and Other Conditions: Supplementary Information

**Sample Evaluation Procedures.**

Evaluation procedures differed based on the original Center for Autism Research (CAR) study participants completed.

**Videoconferencing sample.** Clinical diagnostic evaluations were conducted through a combination of virtual and in-person procedures in response to the COVID-19 pandemic. Participants were recruited through two studies at the Center for Autism Research: the Social Coordination Study and the Adolescent Communication of Emotion Study (ACES).

*Social Coordination Study.* Participants and their parent(s) completed a self- and parent-report computerized KSADS (1), semi-structured interviews, and standardized questionnaires, including the Social Responsiveness Scale, Second Edition (SRS-2) (2), Social Communication Questionnaire (SCQ) Lifetime Form (3), Behavior Assessment System for Children-Third Edition (BASC-3) (4), Conners 3rd Edition (Conners 3) (5), MASC (6), and Vineland Adaptive Behavior Scales, Third Edition (VABS-3) (7). All participants were administered the two-subtest Wechsler Abbreviated Scale of Intelligence–Second Edition (WASI-II) (8) to estimate full-scale IQ, and subtests from the CELF-5 (9) as a screener of language performance. Record review was also conducted. When clinical concern for autism was present and an additional behavioral sample was needed for diagnostic clarity, activities from the Autism Diagnostic Observation Schedule 2 (ADOS-2) (10), Module 3 were administered. Clinicians (supervised by a licensed clinical psychologists) incorporated all available information to determine final diagnoses using clinical best estimate. Clinicians considered all DSM-5 diagnoses for which information was available. N = 238 participants.

*ACES.* Participants and their parents completed the Mini-International Neuropyschiatric Interview for Children and Adolescents (MINI-KID) computerized interview, semi-structured interviews, the ADOS-2, and questionnaires, including the SRS-2, SCQ, VABS-3, and Screen for Child Anxiety Related Emotional Disorders (SCARED) (11). N = 142 participants.

For both the Social Coordination and ACES studies, participants were assigned to the AUT group if they currently met criteria for autism spectrum disorder (regardless of whether they also met criteria for other DSM-5 disorders), the PSY group if they currently met criteria for any DSM-5 disorder except autism spectrum disorder, and the NT group if they did not meet diagnostic criteria for any DSM-5 disorder.

**Face-to-face sample.** Participants in the face-to-face sample were evaluated through a pilot study at CAR, or through the Social Coordination study (pre-pandemic). Evaluation procedures for the face-to-face participants in the Social Coordination study (N = 21) were similar to those described above, except that all evaluation procedures were conducted in person, and all participants were administered the ADOS-2 Module 3.

*Face-to-face pilot study.* Evaluation procedures included administration of the Autism Diagnostic Observation Schedule (ADOS-2), Module 3 or 4, brief unstructured interviews, record review, and parent- and self-report questionnaires, including SRS-2, SCQ, VABS-3, SCARED (parent), and Child Behavior Checklist (CBCL) (12). Autism diagnoses were confirmed through the best clinical judgment of a licensed psychologist using all available information. Of note, because this study did not have an explicit goal of recruiting a mixed psychiatric sample, clinicians did not comprehensively evaluate all DSM-5 conditions. When other conditions were apparent (i.e., through record review, report, or observation) and sufficient evidence was available to provide clinical confidence, clinicians diagnosed other conditions. Individuals were classified as AUT if they currently met criteria for autism spectrum disorder (regardless of whether they also met criteria for other DSM-5 disorders) and NT if they did not receive a diagnosis of any DSM-5 disorder. Participants who received another diagnosis (e.g., anxiety, ADHD), were excluded from the present study. N = 208.

**Cognitive assessment**

Cognitive ability was assessed for each participant with one of: the two- or four-subtest Estimated IQ from the Wechsler Abbreviated Scale of Intelligence, Second Edition(8), Full Scale IQ from the Wechsler Intelligence Scale for Children, Fifth Edition(13), Brief IQ from the Stanford Binet Intelligence Scales, Fifth Edition(14), or General Conceptual Ability from the Differential Abilities Scale, Second Edition(15).

**Inclusion and Exclusion Criteria**

Inclusion criteria for all participants in the original studies included English as a primary language. Exclusion criteria for all participants in the original studies included a known disorder, injury, or medication use which affects motor functioning. For the present study, additional selection criteria included the availability of a video of the primary experimental task (the CASS) with working audio and video, which was able to be processed with 3DI. Individuals were excluded from the PSY and NT groups if they had a first-degree relative with autism spectrum disorder (individuals in the AUT group could have a first-degree relative with autism).

**Concurrence Method: Robustness of results to window length selection**

Coordination was calculated using thirteen segment sizes (i.e., time windows) from 2-32 seconds to explore the effect of the time window selection on the magnitude of group differences. For each time window, concurrence was computed ten times with random parameter initializations and averaged per-person. Within the videoconferencing sample, the maximal group differences were observed at 5 seconds for AUT vs. NT and PSY vs. NT, and 4 seconds for AUT v. PSY. The maximal AUT vs. NT difference was observed at 24 seconds in the face-to-face sample (both the adolescent sample and the full age sample). Notably, the effect sizes across a large range of time windows (~4-28 seconds) were fairly stable.


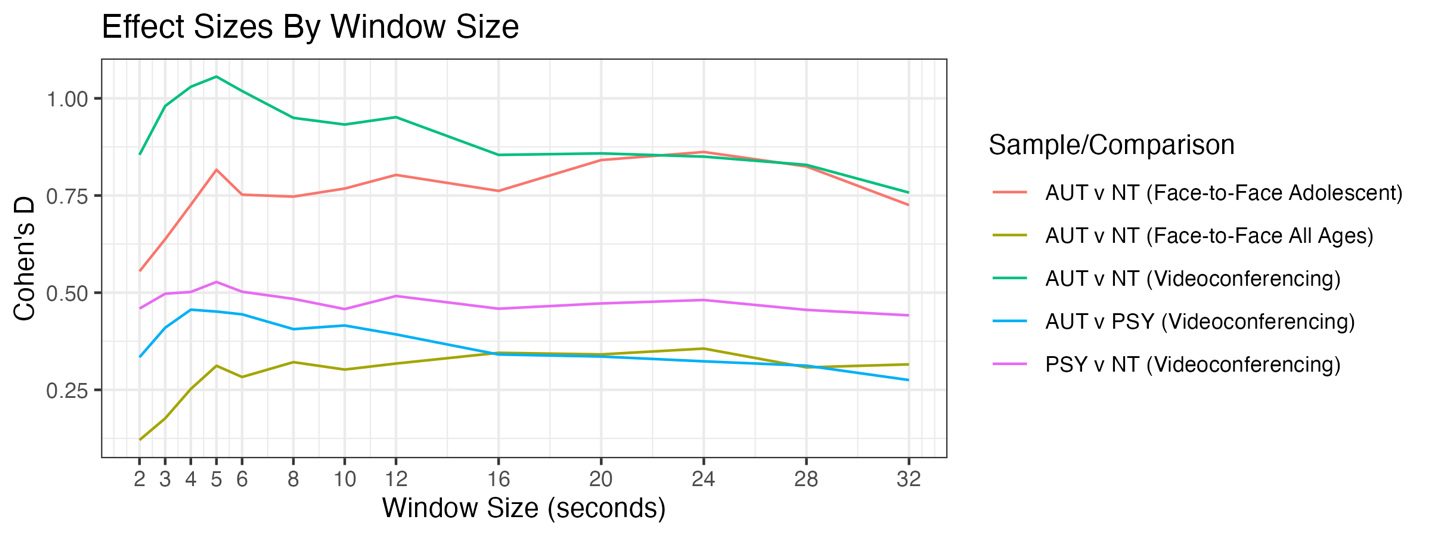


Figure S1 – The effect of the choice of the time window for group analyses (i.e., effect size). For each time window, we plot the effect size (Cohen’s D) of the specified group comparison within the specified sample.

|  |
| --- |

**Imitation Score Generation**

Participants who completed the imitation task imitated 6 movements demonstrated on a video display, with each movement repeated 4 times consecutively, and the entire series repeated in a second trial. For each of the 6 movements from each of the two trials, three raters viewed the video of the participant side-by-side with the video of the model and rated overall accuracy on a 1 (not accurate at all) to 4 (very accurate) scale. For each movement, ratings were averaged across the three raters, resulting in 6 movement scores x 2 trials. For each trial, PCA was used to create a single imitation score across the movements. Trial 2 imitation scores were used, as these previously showed larger AUT/NT group differences than trial 1 imitation scores (16).

**Conversation Rating Scale Versions**

Two versions of the Conversation Rating Scale were adapted from (17).

15-item version used in videoconferencing sample:

1. I felt comfortable with the other person.
2. I liked talking to the other person.
3. It was easy to talk to the other person.
4. I felt “in sync” with the other person.
5. The conversation flowed smoothly.
6. The other person was interested in what I had to say.
7. The other person was nice and friendly.
8. I had to work hard to keep the conversation going.
9. The conversation felt awkward.
10. The other person seemed bored by our conversation.
11. The other person seemed disconnected.
12. The other person made good eye contact with me.
13. The other person looked comfortable.
14. The other person’s face showed how they were feeling.
15.
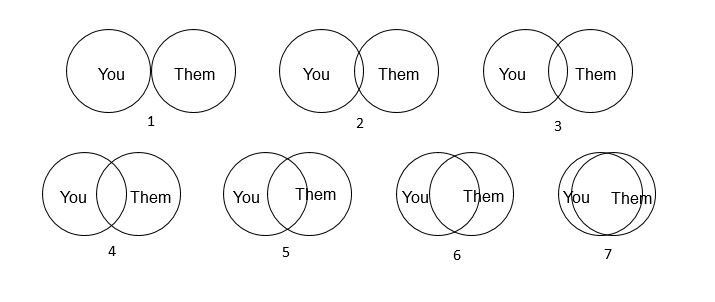
After talking to the other person, choose the picture that best shows how close you feel to them.

6-item version used in face-to-face sample:

1. The other person was interested in what I had to say
2. This person was warm and friendly.
3. The conversation flowed smoothly.
4. The other person acted bored by our conversation.
5. The other person created a sense of distance between us.
6. The other person made appropriate eye contact with me during conversation.

**Matched videoconferencing and face-to-face samples**

Table S1. Participant characteristics of the sample created by matching across the videoconferencing and face-to-face samples on group, sex, age, and IQ.

|  | **AUT (N=91)** | **NT (N=47)** | **P-value** |
| --- | --- | --- | --- |
| **Age** |  |  |  |
| Mean (SD) | 15.2 (1.99) | 14.2 (1.76) | 0.00308 |
| Median [Min, Max] | 14.9 [12.0, 18.7] | 13.6 [12.1, 18.0] |  |
| **Sex** |  |  |  |
| Female | 22 (24.2%) | 23 (48.9%) | 0.00598 |
| Male | 69 (75.8%) | 24 (51.1%) |  |
| **IQ** |  |  |  |
| Mean (SD) | 96.3 (18.8) | 107 (10.3) | <0.001 |
| Median [Min, Max] | 99.0 [47.0, 136] | 106 [86.0, 136] |  |
| **SRS-2** |  |  |  |
| Mean (SD) | 68.7 (11.3) | 44.6 (5.35) | <0.001 |
| Median [Min, Max] | 69.0 [45.0, 91.0] | 43.0 [38.0, 60.0] |  |
| Missing | 18 (19.8%) | 12 (25.5%) |  |
| **Set-up** |  |  |  |
| face-to-face | 46 (50.5%) | 23 (48.9%) | 1 |
| videoconferencing | 45 (49.5%) | 24 (51.1%) |  |

|  | **face-to-face (N=69)** | **videoconferencing (N=69)** | **P-value** |
| --- | --- | --- | --- |
| **Age** |  |  |  |
| Mean (SD) | 14.9 (2.02) | 14.8 (1.93) | 0.79 |
| Median [Min, Max] | 14.1 [12.0, 18.7] | 14.4 [12.1, 18.3] |  |
| **Sex** |  |  |  |
| Female | 23 (33.3%) | 22 (31.9%) | 1 |
| Male | 46 (66.7%) | 47 (68.1%) |  |
| **IQ** |  |  |  |
| Mean (SD) | 99.4 (20.7) | 100 (12.8) | 0.726 |
| Median [Min, Max] | 103 [47.0, 136] | 100 [76.0, 135] |  |
| **SRS-2** |  |  |  |
| Mean (SD) | 59.6 (12.5) | 61.6 (16.2) | 0.469 |
| Median [Min, Max] | 60.0 [39.0, 81.0] | 63.0 [38.0, 91.0] |  |
| Missing | 30 (43.5%) | 0 (0%) |  |
| **Group** |  |  |  |
| AUT | 46 (66.7%) | 45 (65.2%) | 1 |
| NT | 23 (33.3%) | 24 (34.8%) |  |


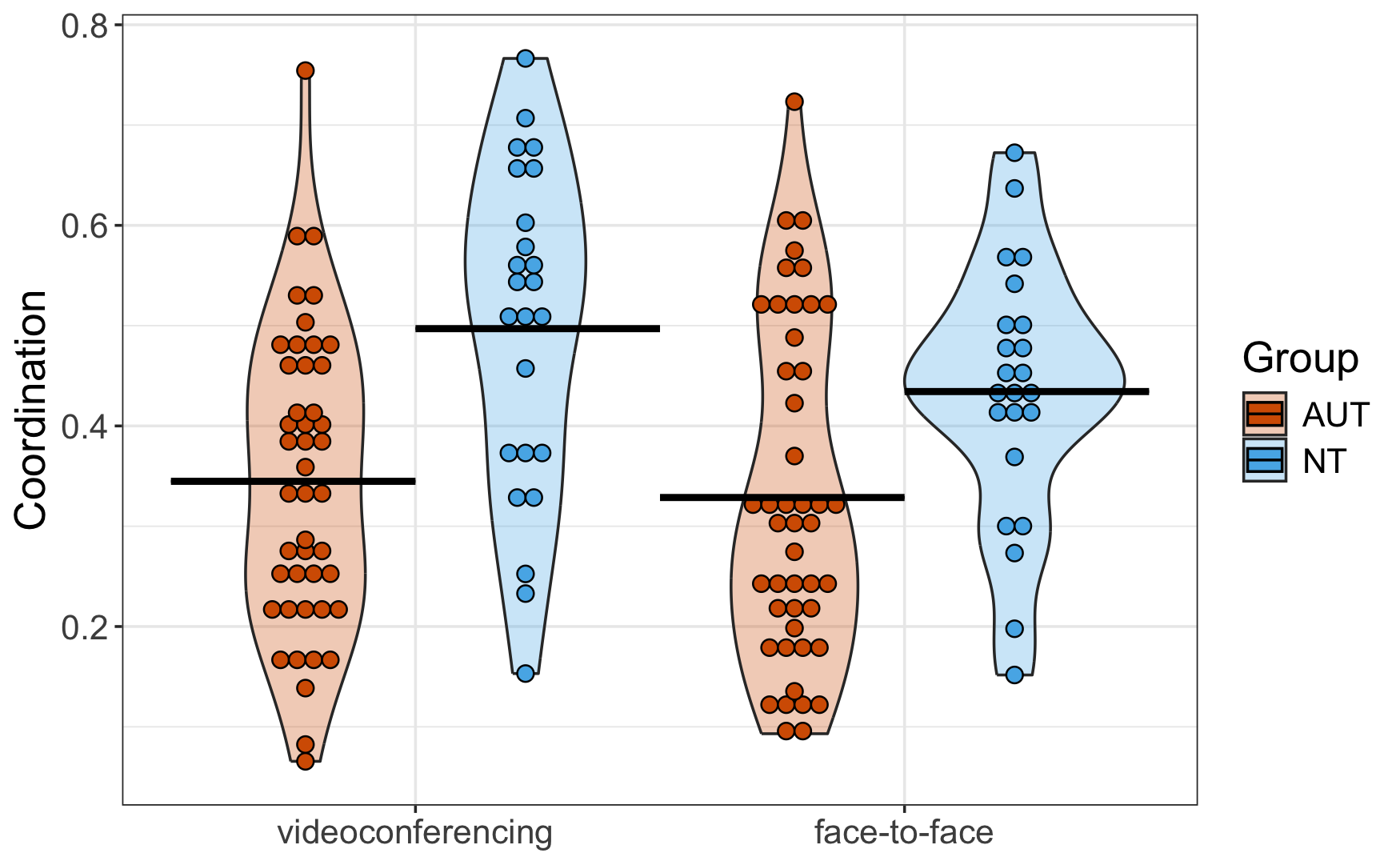


Figure S2. Coordination scores in samples of videoconferencing and face-to-face participants matched on proportion of AUT/NT, sex, age, and IQ.

**Face-to-face sample: full age range**

Table S2. Participant characteristics of the full sample of face-to-face participants, with no age restriction. Our replication analyses focus on 12-18 year-olds to align with the age range of the videoconferencing sample (characteristics presented in main Table 1).

|  | **AUT (N=108)** | **NT (N=121)** | **P-value** |
| --- | --- | --- | --- |
| **Age** |  |  |  |
| Mean (SD) | 16.2 (7.63) | 17.8 (12.4) | 0.216 |
| Median [Min, Max] | 14.1 [7.25, 49.5] | 12.7 [5.53, 52.3] |  |
| **Sex** |  |  |  |
| Female | 31 (28.7%) | 49 (40.5%) | 0.0837 |
| Male | 77 (71.3%) | 72 (59.5%) |  |
| **IQ** |  |  |  |
| Mean (SD) | 98.5 (20.5) | 112 (12.1) | <0.001 |
| Median [Min, Max] | 102 [47.0, 137] | 112 [86.0, 138] |  |
| Missing | 0 (0%) | 15 (12.4%) |  |
| **SRS-2** |  |  |  |
| Mean (SD) | 66.4 (9.87) | 46.0 (6.01) | <0.001 |
| Median [Min, Max] | 68.0 [47.0, 89.0] | 45.0 [38.0, 65.0] |  |
| Missing | 29 (26.9%) | 27 (22.3%) |  |
| **Race** |  |  |  |
| Asian | 3 (2.8%) | 7 (5.8%) | 0.00197 |
| Black or African American | 8 (7.4%) | 20 (16.5%) |  |
| More than one race | 7 (6.5%) | 8 (6.6%) |  |
| Other | 2 (1.9%) | 0 (0%) |  |
| Unknown/Not Reported | 3 (2.8%) | 17 (14.0%) |  |
| White | 85 (78.7%) | 68 (56.2%) |  |
| American Indian or Alaska Native | 0 (0%) | 1 (0.8%) |  |
| **Ethnicity** |  |  |  |
| Hispanic or Latino | 9 (8.3%) | 1 (0.8%) | <0.001 |
| Not Hispanic or Latino | 78 (72.2%) | 72 (59.5%) |  |
| Unknown/Not Reported | 21 (19.4%) | 48 (39.7%) |  |

When the full available age range (5-52 years) of face-to-face participants is examined, there is not a significant main effect of diagnosis. However, unlike within the adolescent samples, there is a significant main effect of age, with coordination increasing with age. These results suggest that coordination scores are relatively stable within adolescence, but should be accounted for when examining a very broad age range.

Table S3. Linear model predicting coordination scores in the full age range of face-to-face participants.

| **Characteristic** | ***B*** | **95% CI** | **P value** |
| --- | --- | --- | --- |
| **Group** |  |  |  |
| AUT | — | — |  |
| NT | 0.03 | -0.01, 0.08 | 0.172 |
| **Sex** |  |  |  |
| Female | — | — |  |
| Male | -0.02 | -0.06, 0.03 | 0.396 |
| **Age** | 0.00 | 0.00, 0.01 | <0.001 |
| **IQ** | 0.00 | 0.00, 0.00 | 0.233 |
| Abbreviation: *B* = unstandardized coefficient CI = Confidence Interval | | | |

| **Characteristic** | **Estimate** | **95% CI** | **P value** |
| --- | --- | --- | --- |
| **Group** |  |  |  |
| AUT | — | — |  |
| NT | 0.03 | -0.01, 0.08 | 0.172 |
| **Sex** |  |  |  |
| Female | — | — |  |
| Male | -0.02 | -0.06, 0.03 | 0.396 |
| **Age** | 0.00 | 0.00, 0.01 | <0.001 |
| **IQ** | 0.00 | 0.00, 0.00 | 0.233 |
| Abbreviation: CI = Confidence Interval | | | |


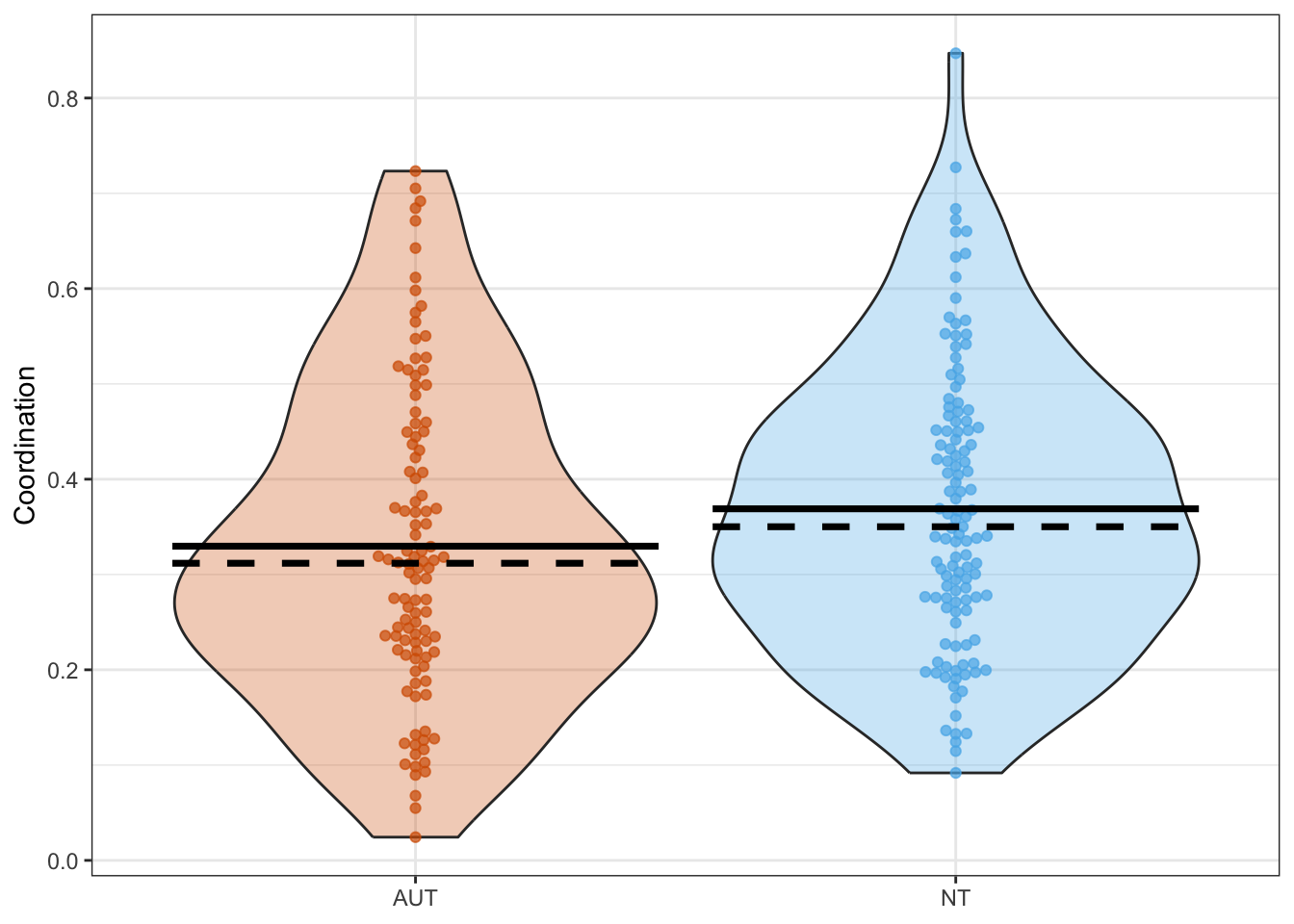


Figure S3. Coordination scores by group for the full age range of face-to-face participants.


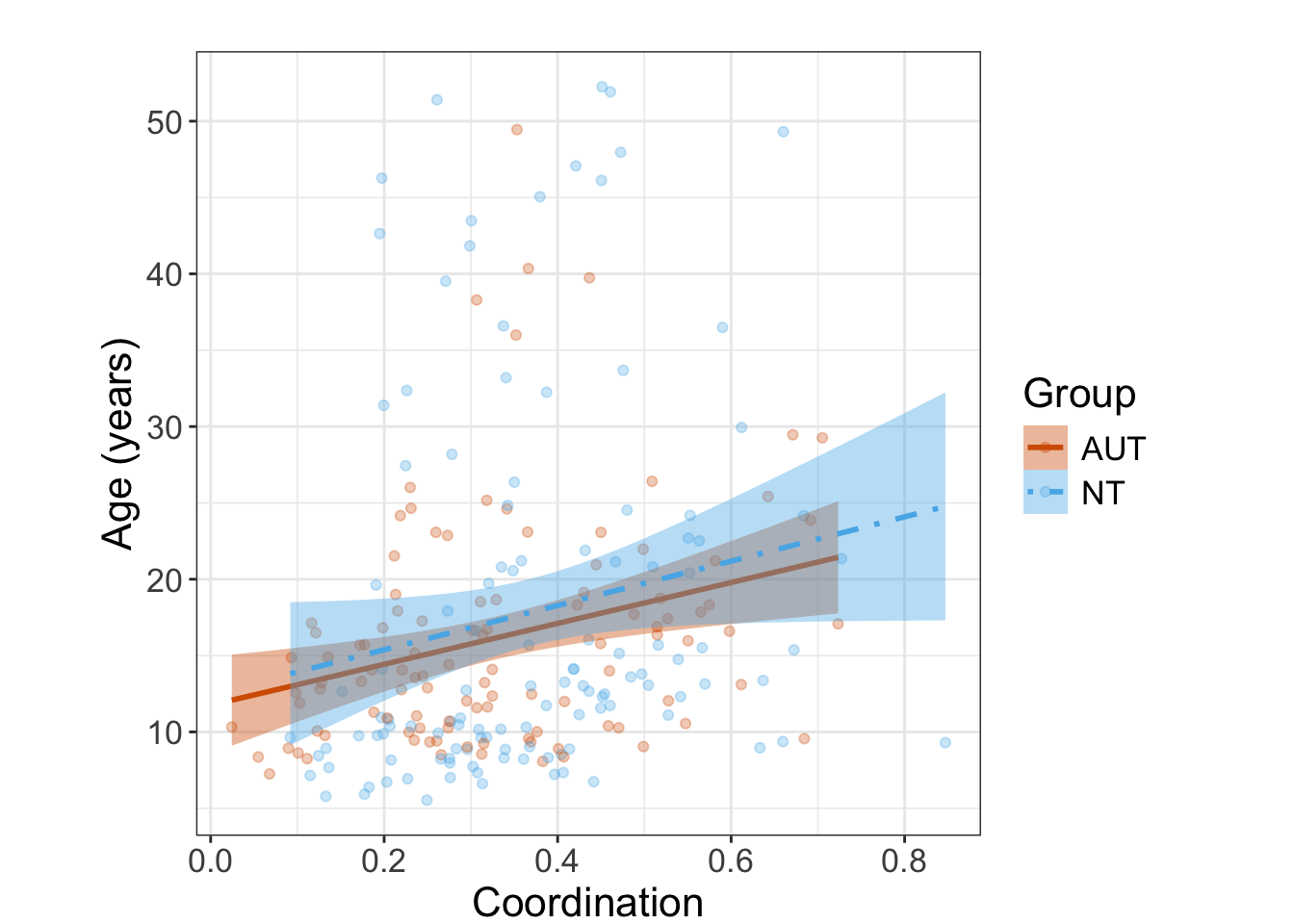


Figure S4. Coordination scores by age (years) for the full age range of face-to-face participants.

**Sensitivity analysis: face-to-face adolescent sample, with first-degree family members removed**

First-degree family members of other participants were not excluded from participation in the pilot study through which face-to-face data were collected. Removing 6 individuals to ensure no first-degree relatives are present in the sample does not change the effect size or significance of the main effect of AUT<NT.

Table S4. Linear model predicting coordination scores in the face-to-face adolescent sample, with first-degree relatives removed.

| **Characteristic** | ***B*** | **95% CI** | **P value** |
| --- | --- | --- | --- |
| **Group** |  |  |  |
| AUT | — | — |  |
| NT | 0.11 | 0.01, 0.20 | 0.033 |
| **Sex** |  |  |  |
| Female | — | — |  |
| Male | 0.01 | -0.07, 0.10 | 0.759 |
| **Age** | 0.02 | 0.00, 0.04 | 0.082 |
| **IQ** | 0.00 | 0.00, 0.00 | 0.209 |
| Abbreviation: *B* = unstandardized coefficient CI = Confidence Interval | | | |

| **Characteristic** | **Estimate** | **95% CI** | **P value** |
| --- | --- | --- | --- |
| **SC_group** |  |  |  |
| AUT | — | — |  |
| NT | 0.11 | 0.01, 0.20 | 0.033 |
| **intake_sex** |  |  |  |
| Female | — | — |  |
| Male | 0.01 | -0.07, 0.10 | 0.759 |
| **age** | 0.02 | 0.00, 0.04 | 0.082 |
| **cog_estimate** | 0.00 | 0.00, 0.00 | 0.209 |
| Abbreviation: CI = Confidence Interval | | | |

Table S5. Linear models predicting expression coordination and pose coordination in the video-conferencing sample.

|  | **Expression Coordination** | | | **Pose Coordination** | | |
| --- | --- | --- | --- | --- | --- | --- |
| **Characteristic** | ***B*** | **95% CI** | **P value** | ***B*** | **95% CI** | **P value** |
| **Group** |  |  |  |  |  |  |
| AUT | — | — |  | — | — |  |
| PSY | 0.07 | 0.03, 0.10 | <0.001 | 0.01 | 0.00, 0.01 | 0.053 |
| NT | 0.14 | 0.10, 0.18 | <0.001 | 0.01 | 0.01, 0.02 | <0.001 |
| **Sex** |  |  |  |  |  |  |
| Female | — | — |  | — | — |  |
| Male | -0.08 | -0.11, -0.05 | <0.001 | 0 | -0.01, 0.00 | 0.139 |
| **Age** | 0.01 | 0.00, 0.02 | 0.048 | 0 | 0.00, 0.00 | 0.067 |
| **IQ** | 0 | 0.00, 0.00 | 0.48 | 0 | 0.00, 0.00 | 0.257 |
| Abbreviation: *B* = unstandardized coefficient CI = Confidence Interval | | | |  |  |  |

Table S6. Linear models predicting expression coordination and pose coordination in the face-to-face adolescent sample.

|  | **Expression Coordination** | | | **Pose Coordination** | | |
| --- | --- | --- | --- | --- | --- | --- |
| **Characteristic** | ***B*** | **95% CI** | **P value** | ***B*** | **95% CI** | **P value** |
| **Group** |  |  |  |  |  |  |
| AUT | — | — |  | — | — |  |
| NT | 0.12 | 0.03, 0.21 | 0.007 | 0.01 | 0.00, 0.02 | 0.066 |
| **Sex** |  |  |  |  |  |  |
| Female | — | — |  | — | — |  |
| Male | 0.01 | -0.07, 0.09 | 0.826 | 0 | -0.01, 0.01 | 0.966 |
| **Age** | 0.02 | 0.00, 0.04 | 0.065 | 0 | 0.00, 0.00 | 0.634 |
| **IQ** | 0 | 0.00, 0.00 | 0.273 | 0 | 0.00, 0.00 | 0.16 |
| Abbreviation: *B* = unstandardized coefficient CI = Confidence Interval | | | |  |  |  |

**References**

1. Townsend L, Kobak K, Kearney C, Milham M, Andreotti C, Escalera J, et al. Development of Three Web-Based Computerized Versions of the Kiddie Schedule for Affective Disorders and Schizophrenia Child Psychiatric Diagnostic Interview: Preliminary Validity Data. Journal of the American Academy of Child & Adolescent Psychiatry. 2020 Feb 1;59(2):309–25. doi:10.1016/j.jaac.2019.05.009

2. Constantino J, Gruber C. The Social Responsiveness Scale Manual, Second Edition (SRS-2). Los Angeles, CA: Western Psychological Services; 2012.

3. Rutter M, Bailey A, Lord C, et al. Social Communication Questionnaire, 2003. Los Angeles, CA: Western Psychological Services; 2003.

4. Reynolds CR, Kamphaus RW, Vannest KJ. BASC3: Behavior Assessment System for Children. PscyhCorp; 2015.

5. Conners CK. Conners 3. In. MHS North Tonawanda, NJ; 2008.

6. March J. MASC 2. Multidimensional Anxiety Scale for Children. 2012.

7. Sparrow S, Cicchetti D, Saulnier C. Vineland adaptive behavior scales–third edition (Vineland-3). Circle Pines, MN: American Guidance Service. 2016.

8. Wechsler D. Wechsler Abbreviated Scale of Intelligence–Second Edition (WASI-II). San Antonio, TX: NCS Pearson; 2011.

9. Wiig EH, Secord WA, Semel E. Clinical evaluation of language fundamentals: CELF-5. Pearson; 2013.

10. Lord C, Rutter M, DiLavore P, Risi S, Gotham K, Bishop S. Autism Diagnostic Observation Schedule, Second Edition: ADOS-2. Western Psychological Services; 2012.

11. Birmaher B, Khetarpal S, Brent D, Cully M, Balach L, Kaufman J, et al. The screen for child anxiety related emotional disorders (SCARED): Scale construction and psychometric characteristics. Journal of the American Academy of Child & Adolescent Psychiatry. 1997;36(4):545–53.

12. Achenbach TM, Rescorla LA. Manual for the ASEBA school-age forms & profiles: child behavior checklist for ages 6-18, teacher’s report form, youth self-report: an integrated system of multi-informant assessment. University of Vermont, research center for children youth & families; 2001.

13. Wechsler D. WISC-V: Technical and interpretive manual. NCS Pearson, Incorporated; 2014.

14. Roid G. Stanford-Binet intelligence scales–fifth edition. Itasca, IL: Riverside. 2003.

15. Elliott CD, Salerno JD, Dumont R, Willis JO. Differential ability scales Second edition. San Antonio, TX. 2007.

16. Zampella CJ, Sariyanidi E, Hutchinson AG, Bartley GK, Schultz RT, Tunç B. Computational Measurement of Motor Imitation and Imitative Learning Differences in Autism Spectrum Disorder. In: Companion Publication of the 2021 International Conference on Multimodal Interaction [Internet]. New York, NY, USA: Association for Computing Machinery; 2021 [cited 2023 Oct 6]. p. 362–70. (ICMI ’21 Companion). Available from: https://dl.acm.org/doi/10.1145/3461615.3485426 doi:10.1145/3461615.3485426

17. Ratto AB, Turner-Brown L, Rupp BM, Mesibov GB, Penn DL. Development of the Contextual Assessment of Social Skills (CASS): A Role Play Measure of Social Skill for Individuals with High-Functioning Autism. J Autism Dev Disord. 2011 Sep 1;41(9):1277–86. doi:10.1007/s10803-010-1147-z
